# Long non-coding RNA DANCR stabilizes HIF-1α and promotes metastasis by interacting with NF90/NF45 complex in nasopharyngeal carcinoma: Erratum

**DOI:** 10.7150/thno.89706

**Published:** 2023-10-15

**Authors:** Xin Wen, Xu Liu, Yan-Ping Mao, Xiao-Jing Yang, Ya-Qin Wang, Pan-Pan Zhang, Yuan Lei, Xiao-Hong Hong, Qing-Mei He, Jun Ma, Na Liu, Ying-Qin Li

**Affiliations:** Sun Yat-sen University Cancer Center; State Key Laboratory of Oncology in South China; Collaborative Innovation Center of Cancer Medicine; Guangdong Key Laboratory of Nasopharyngeal Carcinoma Diagnosis and Therapy, No. 651 Dongfeng Road East, Guangzhou 510060, People's Republic of China

The authors regret to find an error in Figure 5C, in which the Transwell invasion image of siDANCR-1+HIF-1α hypoxic (+) group was mistakenly used. The authors have revised the image of Figure 5C, and confirm that the correction has no effect on the conclusions of the study. Please accept our sincere apologies for any inconvenience caused. Following is a revised arrangement of Figure 5C.

## Figures and Tables

**Figure 5:**
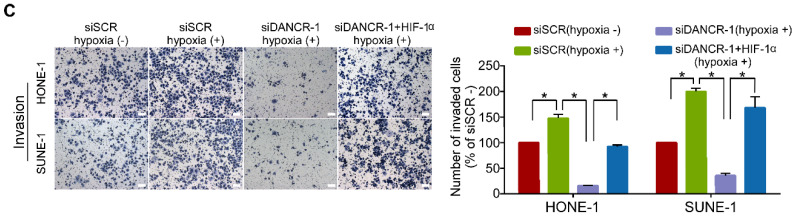
** LncRNA DANCR promotes NPC cell migration and invasion via HIF-1α. (C)** Representative and quantification results of the invasion assays for HONE-1 and SUNE-1 cells under hypoxic conditions. Scale bar, 100 μm. Data are presented as mean ± SD; Student's *t*-tests; *P<0.05.

